# Supplementary material for: Trans-illumination intestine projection imaging of intestinal motility in mice
Source: Nat Commun. 2021 Mar 16;12:1682. doi: 10.1038/s41467-021-21930-w (PMC7966380; doi:10.1038/s41467-021-21930-w)
Supplement: Supplementary file 1 — Supplementary Information [file 41467_2021_21930_MOESM1_ESM.pdf]

Trans-illumination Intestine Projection imaging of intestinal motility in mice

Depeng Wang[1,2], Huijuan Zhang[1], Tri Vu[1], Ye Zhan[1], Akash Malhotra[3], Pei Wang[3], Upendra Chitgupi[1], Aliza Rai[3], Sizhe Zhang[1], Lidai Wang[3], Jan D. Huizinga[4], Jonathan F. Lovell[1] and Jun Xia[1*]

[1]Department of Biomedical Engineering, University at Buffalo, State University of New York, Buffalo, USA;

[2]Department of Biomedical Engineering, Duke University, Durham, USA;

[3]Department of Mechanical and Biomedical Engineering, City University of Hong Kong, Hong Kong, China;

[4]Farncombe Family Digestive Health Research Institute, Department of Medicine, McMaster University, Ontario, Canada

[*]To whom correspondence should be addressed. E-mail: junxia@buffalo.edu

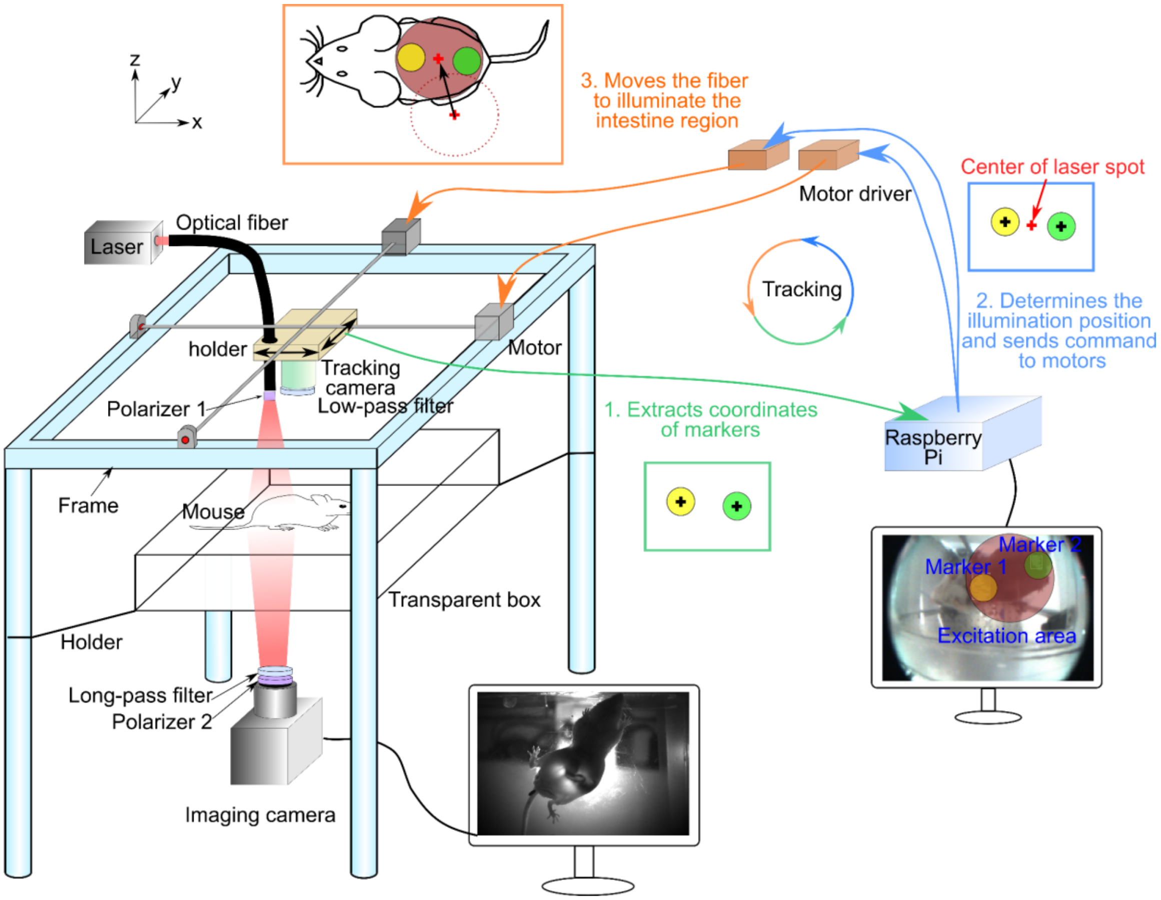

Supplementary Figure 1: Schematic drawing of free-moving imaging system.

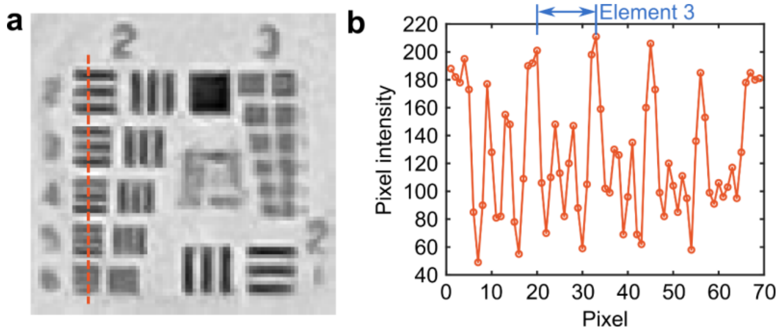

Supplementary Figure 2 Resolution quantification using USAF target demonstrated that 2D-TIP offered a lateral resolution of 99.2 µm. (a) The image of USAF target captured with 2D-TIP. (b) The intensity profile along the dash line in (a) shows that 2D-TIP clearly resolved element in group 2.

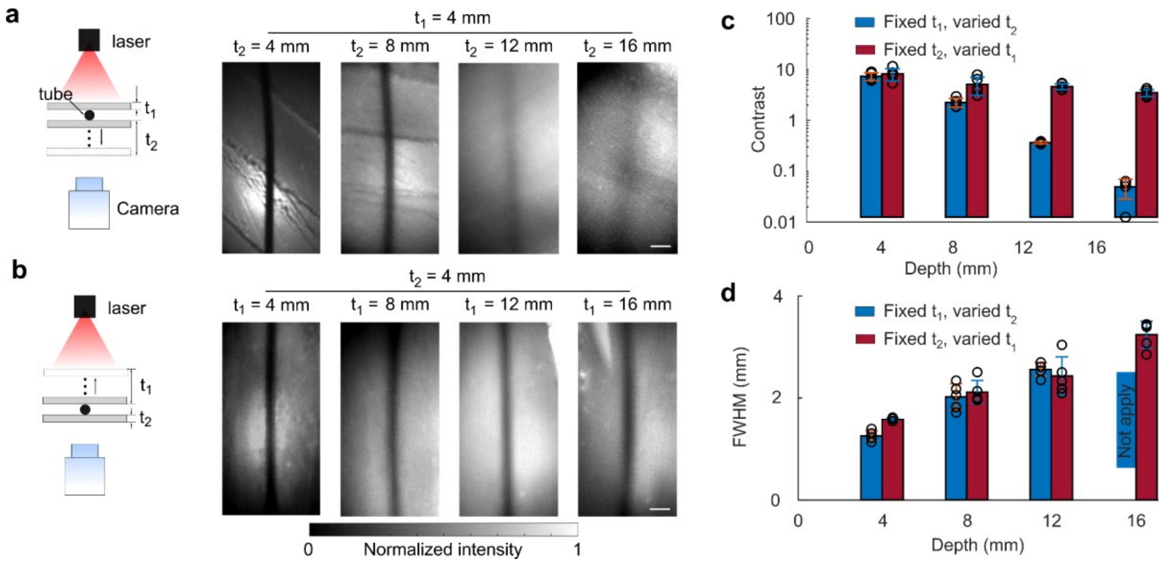

Supplementary Figure 3: System characterization with a 0.5-mm-diameter tube. (a) The tube was imaged in the fixed t_1 mode and the tube blurred quickly as the thickness of t_2 increases. Scale bar: 5 mm. (b) The tube was imaged in the fixed t_2 mode and the tube did not blur significantly as the thickness of t_1 increased. Scale bar: 5 mm. (c) The contrast of tubes imaged in the fixed t_1 and fixed t_2 modes. The contrast of tubes imaged in the fixed t_1 mode degrades quickly but that of the tubes imaged in the fixed t_2 mode only decreased slightly, because the scattering and the absorbance below the tube were fixed, (n =5 measurements, mean ± s.d.). (d) The FWHM of tubes imaged in the fixed t_1 mode increased more than that of the tubes imaged in the fixed t_2 mode, (n =5 independent experiments, mean ± s.d.). When t_2 is 16 mm, the image is too blurry to quantify the FWHM.

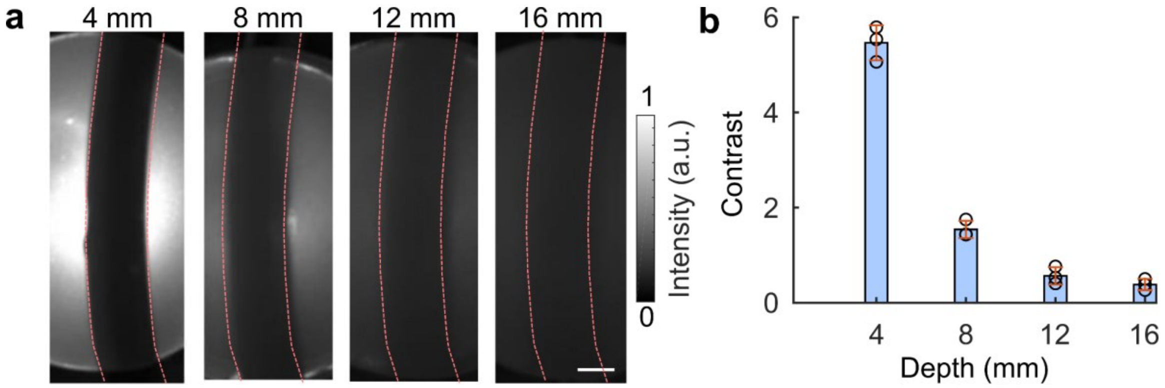

Supplementary Figure 4: The imaging of 4-mm-diameter tube in the fixed t_1 mode showed similar results as the small tube imaging. (a) Images of tube when different thicknesses of agar pieces were placed above tube. When the thickness of the agar piece above the tube is 4 mm, we could clearly see the tube's profile. As the depth further increases, the profile gradually becomes blurrier due to the increased scattering of the agar phantom. Scale bar 3 mm. (b) Contrasts of the tube decreased quickly over the increased thickness of the agar pieces above the tube, (n =3 independent experiments, mean ± s.d.).

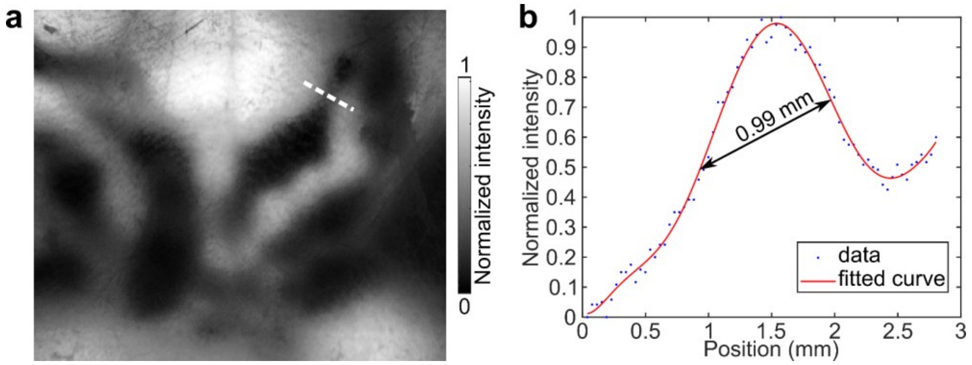

Supplementary Figure 5 TIP identified the contracted intestine. (a) Image of the intestine. The dashed line labeled the cross-section of the contracted intestine. (b) The distribution of the intensity profile of the labeled intestine cross-section in (a). The curve is flipped upside down for display purposes. The contracted intestine only covered around 25% of the intestine diameter.

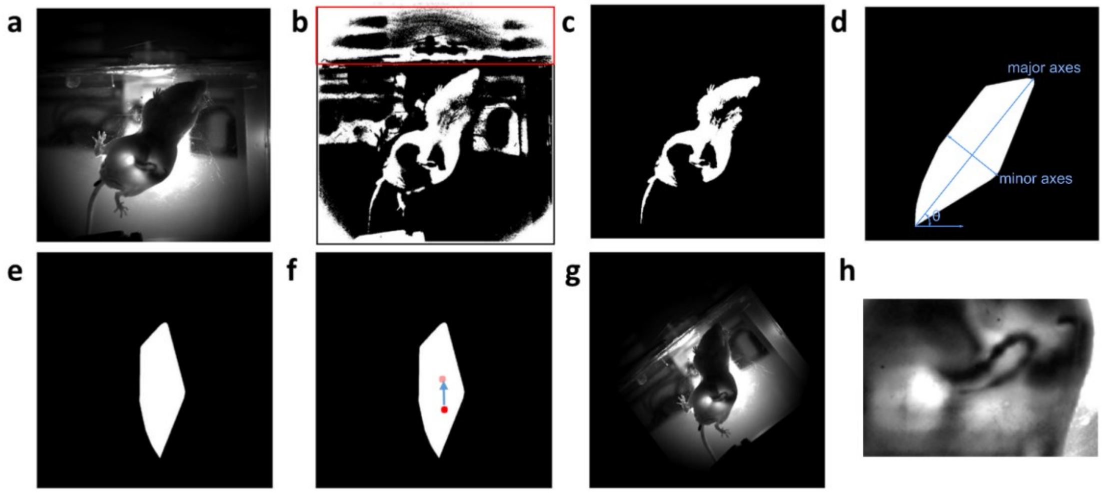

Supplementary Figure 6: Image registration framework. (a) The original video frame before image registration. (b) The complemented binary image shows the top two silhouettes (largest: white region within the red box; second largest: the mice region). (c) The silhouette of the mouse is segmented by placing the second-largest silhouette into the frame. (d) The convex hull of the silhouette. (e) The rotated frame that stands upright. (f) The rotated frames that show the vector (blue arrow) used to fix the intestinal region. The vector is calculated from the anchor point (pink dot: the centroid of the silhouette in the last frame) and the centroid of the silhouette in the current frame (red dot). (g) The registered video frame. (h) The registered and cropped ROI.

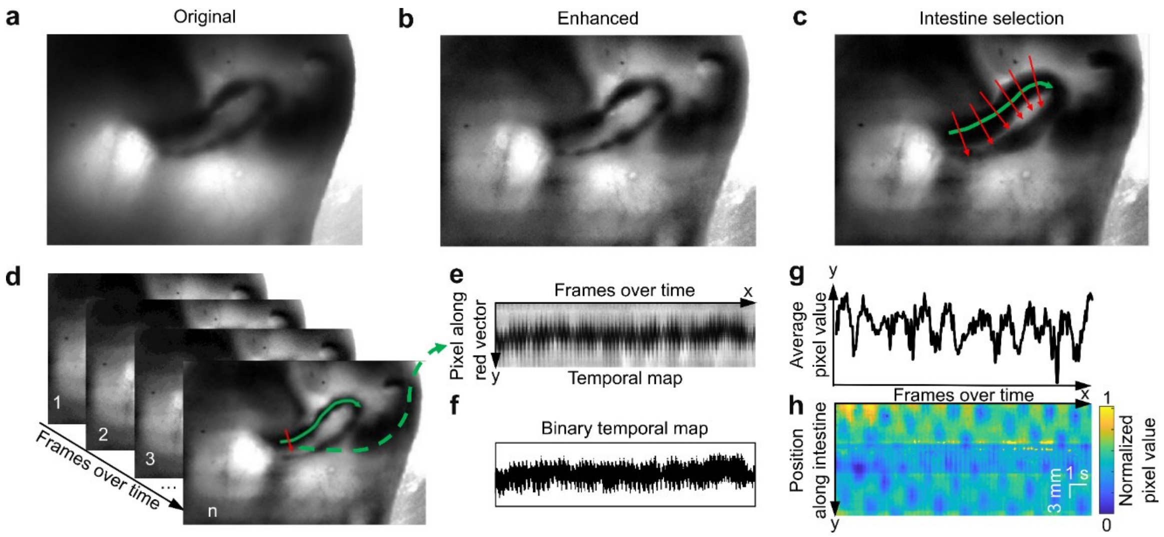

Supplementary Figure 7: Calculation of the spatial-temporal map. (a) The original intestine image. (b) The enhanced intestine image. (c) The MAP image of the enhanced video showing the intestinal region of interest (tracked with a green line). The vertical vectors along the intestine are marked by red arrows. (d) Illustration of signal extraction along a red arrow. (e) The extracted signal profile across all frames. (f) The binary image of the temporal map in (e). (g) The average value of the pixels along the red arrow in (d). (h) Spatial-temporal map of the intestine along the green line in (c).

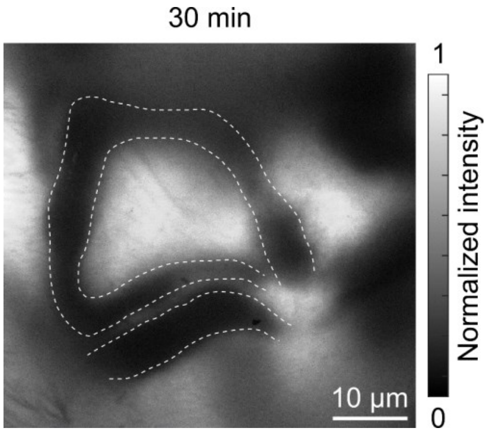

Supplementary Figure 8: TIP revealed intestine structure 30 min post the gavage.

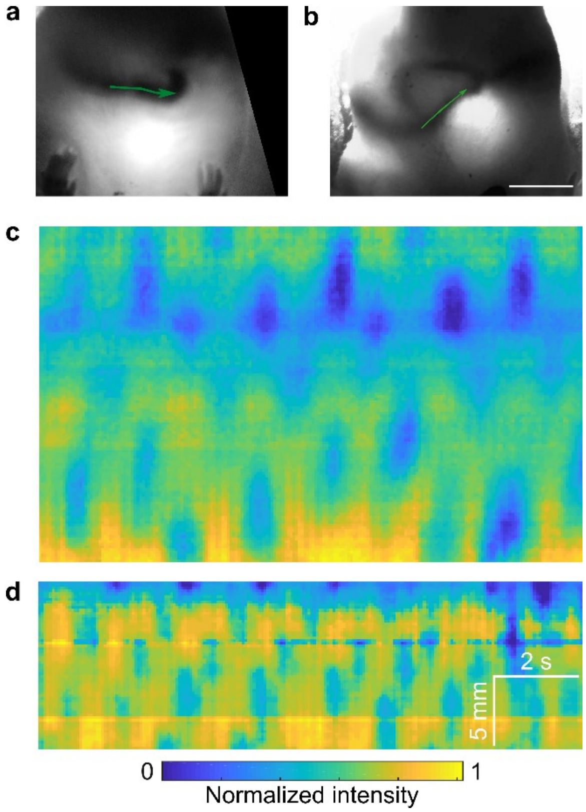

Supplementary Figure 9: Experimental results of additional free-moving mice. (a) Intestine image of one mouse (green line shows the selected intestine). (b) Intestine image of another mouse, scale bar: 10mm. (c) The spatiotemporal map of the mouse in (a) shows the segmentation motor pattern. (d) The spatiotemporal map of the mouse in (b) shows the segmentation motor pattern.

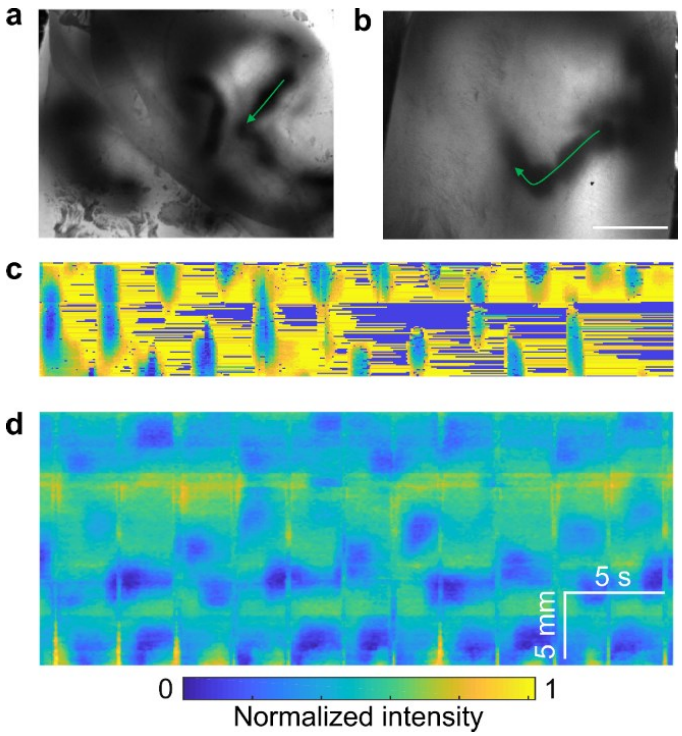

Supplementary Figure 10: Experimental results of additional anesthetized mice. (a) Intestine image of one mouse (green line shows the selected intestine). (b) Intestine image of another mouse, scale bar: 10mm. (c) The spatiotemporal map of the mouse in (a) shows the segmentation motor pattern. (d) The spatiotemporal map of the mouse in (b) shows the segmentation motor pattern.

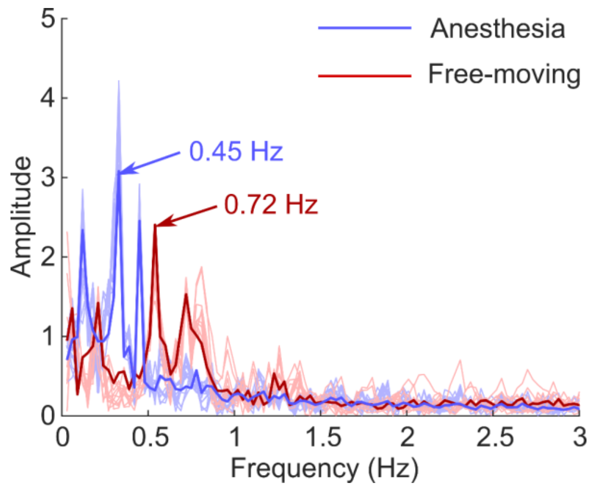

Supplementary Figure 11: Frequency analysis of the segmentation pattern indicates that a free-moving mouse has a higher averaged motility rate than that of an anesthetized mouse. The peak frequency of each state was labeled.

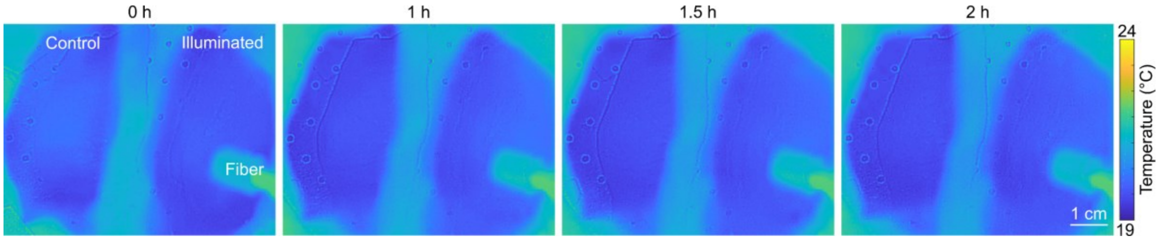

Supplementary Figure 12: Thermal images of the control and illuminated chicken breast tissue over 2 hours. The laser power used in the study did not induce temperature rise in the illuminated chicken breast tissue when compared with the control.

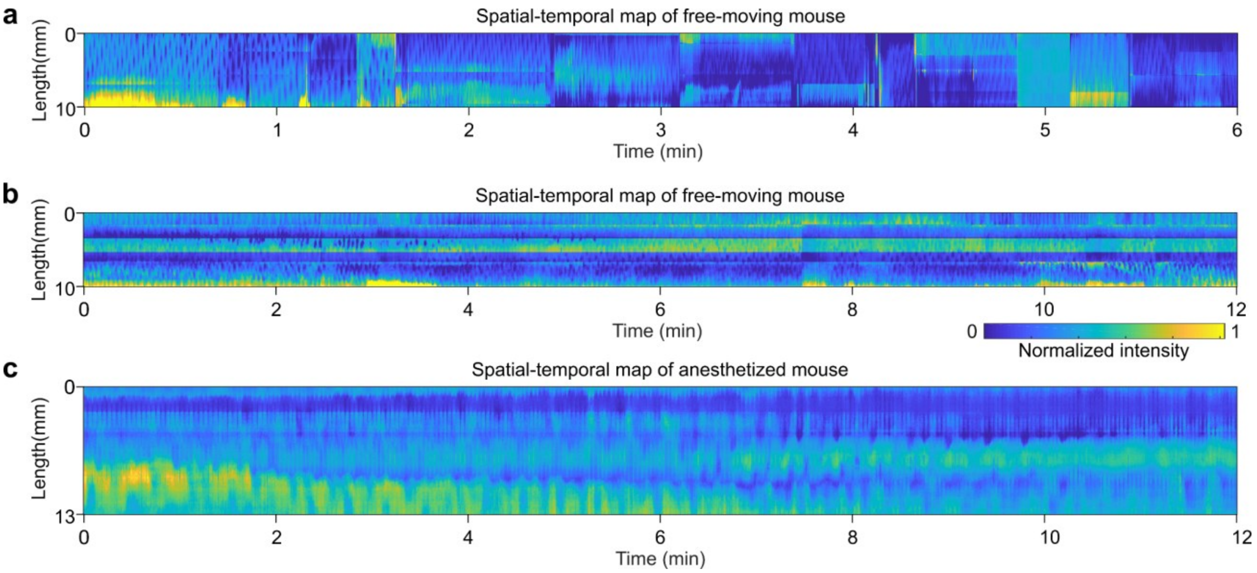

Supplementary Figure 13: (a) A combined pattern for mouse with different behaviors over 6 minutes. Longer duration spatial-temporal map for (b) free-moving mouse and (c) anesthetized mouse.

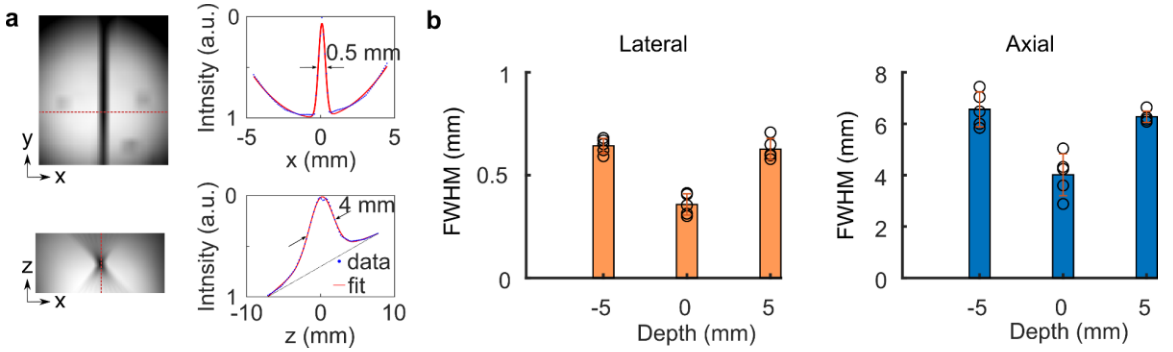

Supplementary Figure 14: Resolution of the 3D-TIP. (a) The quantified FWHM of a black hair in the lateral (top) and axial (bottom) directions. 3D-TIP offers a lateral resolution of 0.5 mm and an axial resolution of 4 mm at 3 mm below the focal plane. (b) Lateral resolution and axial resolution of 3D-TIP vary over depths and are peaked at the focal plane, (mean ± s.d. for $n = 5$ independent experiments).

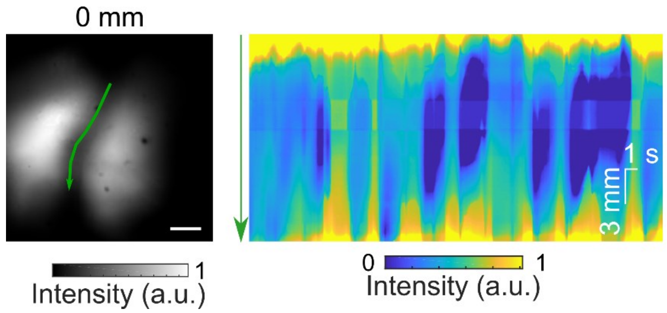

Supplementary Figure 15: 3D-TIP images of mouse intestine at two hours post gavage. The spatial-temporal map shown in the right exhibits peristalsis. Scale bar: 5 mm.

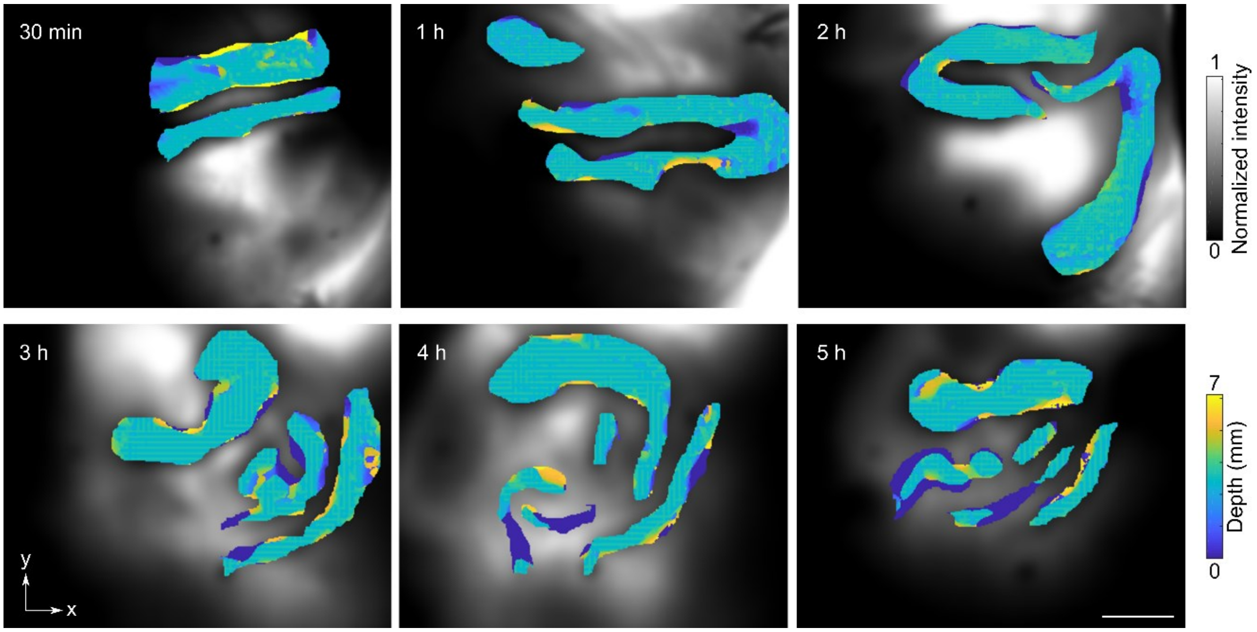

Supplementary Figure 16: Long-duration 3D-TIP imaging of intestine allowed the generation of intestinal map with depth information. The gray images show the intestine profile and the color images show the depth index of the intestine. Scale bar: 7 mm.
