# Supplementary material for: Trans-illumination intestine projection imaging of intestinal motility in mice
Source: Nat Commun. 2021 Mar 16;12:1682. doi: 10.1038/s41467-021-21930-w (PMC7966380; doi:10.1038/s41467-021-21930-w)
Supplement: Supplementary file 3 — Description of Additional Supplementary Files [file 41467_2021_21930_MOESM3_ESM.pdf]

Description of Additional Supplementary Files

File Name: Supplementary Movie 1
Description: Intestine dynamics of free-moving mice 21 min post gavage

File Name: Supplementary Movie 2
Description: Intestine dynamics of free-moving mice 70 min post gavage

File Name: Supplementary Movie 3
Description: Intestine dynamics of free-moving mice 2.5 h post gavage

File Name: Supplementary Movie 4
Description: Intestine dynamics of anesthetized mouse

File Name: Supplementary Movie 5
Description: Three dimensional intestine dynamics
