# Supplementary material for: Trans-illumination intestine projection imaging of intestinal motility in mice
Source: Nat Commun. 2021 Mar 16;12:1682. doi: 10.1038/s41467-021-21930-w (PMC7966380; doi:10.1038/s41467-021-21930-w)
Supplement: Supplementary file 9 — Reporting Summary [file 41467_2021_21930_MOESM9_ESM.pdf]

nature research

Corresponding author(s): Jun Xia

Last updated by author(s): Feb 16, 2021

Reporting Summary

Nature Research wishes to improve the reproducibility of the work that we publish. This form provides structure for consistency and transparency in reporting. For further information on Nature Research policies, see Authors & Referees and the Editorial Policy Checklist.

Statistics

For all statistical analyses, confirm that the following items are present in the figure legend, table legend, main text, or Methods section.

n/a	Confirmed	
☐	☒	The exact sample size (*n*) for each experimental group/condition, given as a discrete number and unit of measurement
☐	☒	A statement on whether measurements were taken from distinct samples or whether the same sample was measured repeatedly
☐	☒	The statistical test(s) used AND whether they are one- or two-sided<br>*Only common tests should be described solely by name; describe more complex techniques in the Methods section.*
☒	☐	A description of all covariates tested
☒	☐	A description of any assumptions or corrections, such as tests of normality and adjustment for multiple comparisons
☐	☒	A full description of the statistical parameters including central tendency (e.g. means) or other basic estimates (e.g. regression coefficient) AND variation (e.g. standard deviation) or associated estimates of uncertainty (e.g. confidence intervals)
☐	☒	For null hypothesis testing, the test statistic (e.g. *F*, *t*, *r*) with confidence intervals, effect sizes, degrees of freedom and *P* value noted<br>*Give P values as exact values whenever suitable.*
☒	☐	For Bayesian analysis, information on the choice of priors and Markov chain Monte Carlo settings
☒	☐	For hierarchical and complex designs, identification of the appropriate level for tests and full reporting of outcomes
☒	☐	Estimates of effect sizes (e.g. Cohen's *d*, Pearson's *r*), indicating how they were calculated

Our web collection on statistics for biologists contains articles on many of the points above.

Software and code

Policy information about availability of computer code

Data collection	We collected data with our TIP system. The details were listed in the Methods section of the manuscript.
Data analysis	Matlab 2017 was used for data analysis and plotting. Inkscape was used to arrange figures. Data analysis is described in detail in the Methods section of the manuscript. Parameters for light-field image reconstruction are extracted from Light Field Display software.

For manuscripts utilizing custom algorithms or software that are central to the research but not yet described in published literature, software must be made available to editors/reviewers. We strongly encourage code deposition in a community repository (e.g. GitHub). See the Nature Research guidelines for submitting code & software for further information.

Data

Policy information about availability of data

All manuscripts must include a data availability statement. This statement should provide the following information, where applicable:

- Accession codes, unique identifiers, or web links for publicly available datasets
- A list of figures that have associated raw data
- A description of any restrictions on data availability

The data that support the findings of this study will be available from the corresponding author upon resonable request.

Field-specific reporting

Please select the one below that is the best fit for your research. If you are not sure, read the appropriate sections before making your selection.

☒ Life sciences ☐ Behavioural & social sciences ☐ Ecological, evolutionary & environmental sciences

October 2018

Life sciences study design

All studies must disclose on these points even when the disclosure is negative.

Sample size	The sample size of each experiment is indicated in the methods and/or figure captions. No sample size calculation is performed. The samples size is chosen based on the reference Zhou, Yang, et al. "A phosphorus phthalocyanine formulation with intense absorbance at 1000 nm for deep optical imaging." Theranostics 6.5 (2016): 688.
Data exclusions	No data were excluded.
Replication	Animal experiments were repeated in three or five independent experiments. Details are provided in the manuscript. All attempts at replication are successful.
Randomization	There was no randomization in these experiment beyond randomly grouping mice in two group without a formal protocol.
Blinding	Blinding was used in our experiments.

Reporting for specific materials, systems and methods

We require information from authors about some types of materials, experimental systems and methods used in many studies. Here, indicate whether each material, system or method listed is relevant to your study. If you are not sure if a list item applies to your research, read the appropriate section before selecting a response.

Materials & experimental systems

n/a	Involved in the study	
☒	☐	Antibodies
☒	☐	Eukaryotic cell lines
☒	☐	Palaeontology
☐	☒	Animals and other organisms
☒	☐	Human research participants
☒	☐	Clinical data

Methods

n/a	Involved in the study	
☒	☐	ChIP-seq
☒	☐	Flow cytometry
☒	☐	MRI-based neuroimaging

Animals and other organisms

Policy information about studies involving animals; ARRIVE guidelines recommended for reporting animal research

Laboratory animals	We used 6-month female ND4 Swiss Webster mice.
Wild animals	This study did not involved wild animals.
Field-collected samples	This study did not involved filed-collected samples.
Ethics oversight	All animal experiments were performed in accordance with the University at Buffalo's Institutional Animal Care and Use Committee.

Note that full information on the approval of the study protocol must also be provided in the manuscript.
